# Interaction between *Helicobacter pylori* and Latent Toxoplasmosis and Demographic Variables on Cognitive Function in Young to Middle-Aged Adults

**DOI:** 10.1371/journal.pone.0116874

**Published:** 2015-01-15

**Authors:** Shawn D. Gale, Lance D. Erickson, Bruce L. Brown, Dawson W. Hedges

**Affiliations:** 1 Department of Psychology, Brigham Young University, Provo, Utah; 2 The Neuroscience Center, Brigham Young University, Provo, Utah; 3 Department of Sociology, Brigham Young University, Provo, Utah; Anhui Medical University, CHINA

## Abstract

*Helicobacter pylori* and latent toxoplasmosis are widespread diseases that have been associated with cognitive deficits and Alzheimer’s disease. We sought to determine whether interactions between *Helicobacter pylori* and latent toxoplasmosis, age, race-ethnicity, educational attainment, economic status, and general health predict cognitive function in young and middle-aged adults. To do so, we used multivariable regression and multivariate models to analyze data obtained from the United States’ National Health and Nutrition Examination Survey from the Centers for Disease Control and Prevention, which can be weighted to represent the US population. In this sample, we found that 31.6 percent of women and 36.2 percent of men of the overall sample had IgG Antibodies against *Helicobacter pylori*, although the seroprevalence of *Helicobacter pylori* varied with sociodemographic variables. There were no main effects for *Helicobacter pylori* or latent toxoplasmosis for any of the cognitive measures in models adjusting for age, sex, race-ethnicity, educational attainment, economic standing, and self-rated health predicting cognitive function. However, interactions between *Helicobacter pylori* and race-ethnicity, educational attainment, latent toxoplasmosis in the fully adjusted models predicted cognitive function. People seropositive for both *Helicobacter pylori* and latent toxoplasmosis – both of which appear to be common in the general population – appear to be more susceptible to cognitive deficits than are people seropositive for either *Helicobacter pylori* and or latent toxoplasmosis alone, suggesting a synergistic effect between these two infectious diseases on cognition in young to middle-aged adults.

## Introduction

Increasing evidence suggests an association between some common infectious diseases and cognitive function in otherwise healthy older adults [1, 2]. Some studies have associated various infectious diseases with dementia, although the results have been inconsistent [3–6]. Katan et al. [1] found that a weighted infectious-disease burden index representing multiple infectious diseases, including *Chlamydia pneumoniae*, cytomegalovirus, *Helicobacter pylori*, and Herpes 1 and 2, predicted decreased cognitive functioning in older adults. However, less work has been done regarding whether these or other infectious diseases might affect cognition in young to middle-aged adults and whether specific infectious diseases may interact with each other in association with cognitive outcome.

In this study, we focus on two widespread infectious diseases, *Helicobacter pylori* and latent toxoplasmosis, and their association with cognitive function. *Helicobacter pylori *(*H. pylori*) is a gram-negative bacterium associated with gastritis and peptic-ulcer disease [7]. *H. pylori* has an estimated prevalence in the U.S. of approximately 25 to 32 percent [8], although the prevalence varies widely between countries, with estimates as high as 80 percent in some countries [8]. A recent study in healthy adults aged 20 to 90 found that *H. pylori* seropositivity was related to cognitive function [9]. The CagA strain of *H. pylori* is particularly pathogenic and could affect the reported association between *H. pylori* infection and cognition, although a recent study did not find evidence of an added effect from CagA on cognition over the presence of *H. pylori* alone [9]. *H. pylori* infection has also been associated with Alzheimer’s disease [5, 10].

Latent toxoplasmosis is caused by *Toxoplasmosis gondii*(*T. gondii*), a protozoal parasite that can infect human brain, eye, and muscle tissue. Although it does not reproduce in humans [11], cysts in muscle and the brain can persist for the life of the host [12]. *T. gondii* infection has an estimated worldwide prevalence of 30 percent and a U.S. prevalence of approximately 22 percent [13] and is one of the most common infections [12]. Once regarded as a benign condition, latent toxoplasmosis in humans has been linked to behavioral and psychiatric abnormalities [14], and cognitive deficits in otherwise healthy individuals [15–17]. A preliminary study suggested a possible association between latent toxoplasmosis and Alzheimer’s disease [18].

In addition to main effects on cognition, infectious diseases may interact with sociodemographic variables. For example, interactions between *H. pylori* and sex and between *H. pylori* and race predicted cognitive function [9]. Similarly, significant interactions between latent toxoplasmosis and educational attainment, economic status, and race-ethnicity predicted cognitive function in young and middle-aged adults [16]. Accordingly, interactions between *H. pylori* and latent toxoplasmosis could affect cognitive function..

Given the reported interactions between *H. pylori* and sex and race predicting cognitive function, the reported associations between latent toxoplasmosis and cognitive function, and the suggestion that infectious diseases may interact to affect cognitive function, we hypothesized that the presence of both *H. pylori* and *T. gondii* would result in worse cognitive function than the presence of either infection alone.

## Method

### Study Sample

Data were from the Centers for Disease Control’s National Health and Nutrition Examination Survey III (NHANES III). We restricted our analysis to participants aged 20 to 59 years because participants aged 12 to 19 years and 60 years and older were evaluated with different cognitive tests than were the participants aged 20 to 59 years. The NHANES III dataset (1988–1994) includes health-related data obtained via questionnaire, clinical examination, and laboratory testing and utilizes a complex sample-weighting method so that the data are representative of the U.S. population [19]. Data in this study were collected by the U.S. government (National Center for Health Statistics which is part of the Centers for Disease Control) in compliance with all federal laws concerning ethical guidelines including obtaining informed consent. Data are anonymized and freely available online.

### Cognitive Function

Cognitive function in NHANES III was assessed with standardized computer-administered testing. The specific tests included simple reaction time (SRT), symbol-digit substitution (SDS), and serial-digit learning (SDL); these tests are broad measures of simple reaction time, processing speed, and memory, respectively. Details regarding the history and development of these tests including descriptions and examples of the stimuli, reliability, and validity can be found elsewhere [20–22]. Briefly, the SRT consists of 50 trials in which the subject is required to make a button press in response to a square displayed mid-screen, which has an inter-stimulus presentation time that randomly varies between 2,500 and 5,000 milliseconds. Response latency (stimulus presentation to button press) was measured in milliseconds. The SDS is a coding task that pairs numbers (1 to 9) with symbols. Subjects see a grid on the top of the screen with the number-symbol pairs and a similar grid with just the symbols below it. In the lower grid, the symbols are in a different order, and subjects are required to enter the numbers to complete the number-symbols pairs as rapidly as possible. Five similar but different trials were completed. The first trial was a practice trial that would not allow incorrect entries. The total amount of time in seconds to complete the remaining 4 trials was the outcome measure. The SDL presents a series of digits one at a time for 600 milliseconds, with a 600 millisecond inter-stimulus interval, after which the subject must enter the complete sequence of digits. The first trial was for practice and included 4 digits. Subsequent trials consisted of an identical 8-digit sequence that is discontinued when the subject scores two consecutive trials correctly or completes a maximum of 8 trials. The score on the SDL is the number of trials to reach criterion up to a maximum of 8. On all of these tests, higher scores represent poorer cognitive performance.

### *H. Pylori* and CagA Status

*H. pylori* and CagA status were represented as three categories—*H. pylori* negative and CagA negative, *H. pylori* positive and CagA negative, and *H. pylori* positive and CagA positive [23]. First, *H*. *pylori* was identified by IgG antibody levels obtained according to NHANES protocols. *H. pylori* antibodies were assayed using *H. pylori* immunoglobulin G (IgG) enzyme-linked immunosorbent assay (Wampole Laboratories, Cranbury, NJ). The optical density for each specimen was divided by the mean optical density of the cutoff controls to identify the immune status ratio. An immune status ratio ranging from 0 to .90 was considered negative, from .91 to 1.09 equivocal, and 1.10 and greater as positive (http://www.cdc.gov/nchs/data/nhanes/nhanes_99_00/lab11_met_helicobacter_pylori.pdf). There were 51 respondents (2.8 percent of the analytic sample) equivocal for *H. pylori* that were treated as negative for the purposes of these analyses. Anti-CagA IgG antibodies were assessed using a non-commercial method developed by Vanderbilt University [24] resulting in an identification of whether the *H. pylori* strain was CagA-positive or negative. The CagA-positive strain of *H. pylori* has been associated with increased inflammation and higher risk for gastric cancer [24]. The *H. pylori* negative and CagA negative group included all respondents negative for both *H. pylori* and the CagA antigen. The *H. pylori* positive and CagA positive group included all subjects positive for the CagA antigen regardless of the results of the *H. pylori* classification. The remaining cases were *H. pylori* positive and CagA negative.

### Latent Toxoplasmosis

Latent toxoplasmosis was determined by comparing the optical density readings of *T. gondii* IgG antibody levels for each sample to a standard curve constructed from positive control sera. The standard curve was calibrated to World Health Organization Toxo 60 serum. The NHANES III laboratory documentation indicates that antibody levels below 7 IU/mL were considered negative for latent toxoplasmosis, whereas levels above 7 IU/mL were considered positive (ftp://ftp.cdc.gov/pub/Health_Statistics/NCHS/nhanes/nhanes3/1A/lab-acc.pdf) (p.122). Subjects were coded 1 for latent toxoplasmosis positive if their antibody levels exceeded 7 IU/mL, and subjects below that level were coded 0 for latent toxoplasmosis negative.

### Demographic Covariates

Gender was coded as 0 for male and 1 for female while age was included as a continuous variable. Information regarding race was self-reported by subjects based on categories provided by NHANES, which included white, black, and other (including Aleut, Eskimo, American Indian, Asian or Pacific Islander, and other). NHANES ethnicity categories were Hispanic (Mexican-American and other Hispanic) and non-Hispanic. We combined these two measures forming a “race-ethnicity” variable that included categories for Non-Hispanic white, Non-Hispanic black, Hispanic (regardless of racial categorization), and other. Level of education was a continuous variable operationalized as the years of education obtained. Poverty-to-income ratio (PIR) was a continuous variable. To obtain the PIR, the respondents’ reported family income was divided by US Census Bureau definition of poverty for the year of the interview. The poverty definition for each respondent was appropriate to their family size. PIR values lower than 1 were considered to be below the poverty threshold, and 1 or greater indicated income to be at or above the poverty level. Preliminary analysis indicated PIR’s relationship with each dependent variable had two distinct linear components. Consequently, we modeled PIR as a spline with the knot or deflection point at PIR of 3. Self-rated health was the respondents’ assessment of their health on a 5-point scale ranging from 1 “poor” to 5 “excellent” and was treated in models as a continuous variable because it was on an ordered scale.

### Control

Hemoglobin concentration was measured according to procedures detailed elsewhere [25]. Given that the presence of *H. pylori* has been associated with anemia [26] and that iron deficiency has been associated with cognitive function [27], we controlled for anemia in the present study by including hemoglobin concentration as a covariate. We used hemoglobin to estimate the presence of anemia because in addition to the association between iron-deficiency anemia and *H. pylori* infection, there may be an association between megaloblastic anemia and *H. pylori* due to vitamin B12 deficiency [28]. Hemoglobin was measured in g/dL and was included in models as a continuous variable.

### Statistical Analyses

We used Stata 13.1 (StataCorp, College Station, Texas) for all statistical analyses. Because of the complex sampling design of NHANES, Stata’s svy command prefix was used for all analyses to include 1) sampling weights so parameter estimates are representative of the U.S. civilian, non-institutionalized population aged 20 to 59 years and 2) information on clustering in the sample selection process to adjust standard-error estimates. Furthermore, because we analyzed three measures of cognitive functioning as outcomes, we estimated multivariate tests to include all three measures of cognitive outcome in the same model. Traditional MANOVA and MANCOVA methods are not available for use with these data because complex sampling designs cannot be taken into account using these methods in Stata or in any of the commonly used statistical packages. We therefore adopted the procedure described by Timm [29] to create multivariate tests for complex sampling data by combining the parameter estimates from the univariate (i.e., a single dependent variable) OLS regressions for the three dependent variables, including the corresponding covariance parameters, into a single vector that can be tested using Stata’s maximum likelihood *suest* command. We followed the procedure recommended by Rencher and Christensen [30] and by Brown, Hendrix, Hedges, and Smith [31] of only considering univariate test results statistically significant (i.e., the relationship between a covariate and a single measure of cognitive functioning) when the corresponding multivariate test is also significant (i.e., the joint relationship defined by the combined pattern on all three measures of cognitive functioning). Rencher and Scott [32] have demonstrated this is an effective way to avoid reporting chance results due to estimating a large number of univariate statistical tests.

Analyses were completed in two stages. First, we predicted cognitive functioning as measured by the SDS, SRT, and SDL using latent *H. pylori* and toxoplasmosis as the main predictors controlling for age, gender, race-ethnicity, education, PIR, and self-rated health. Second, we estimated an additional series of models for each measure of cognitive functioning to explore interactions of *H. pylori* with latent toxoplasmosis and the control variables. Each model included all control variables and an interaction between *H. pylori* and latent toxoplasmosis or one of the control variables.

## Results

The overall seroprevalence by gender of *H. pylori* was 31.6% for females and 36.2% for males. Weighted prevalences of *H. pylori* and CagA infection across several demographics from the NHANES sample are presented in Table 1. The prevalence of *H. pylori* and CagA was significantly higher in people also positive for latent toxoplasmosis. Among the demographic covariates, there were significant differences in prevalence according to age, race-ethnicity, education, PIR, and self-rated health. In particular, prevalence increased with age, was lower in non-Hispanic Whites than non-Hispanic blacks, Hispanics, and other race-ethnicities, decreased with increasing educational attainment, was higher in people living in poverty than those not living in poverty, and decreased with increasing ratings of self-reported health. There were no significant differences in *H. pylori* and CagA prevalence across tertiles of hemoglobin.

**Table 1:** Weighted Prevalence of *Helicobacter pylori* Across Demographic Characteristics in US 21 to 59 Year-Olds, NHANES III (1988–1994).

			***H. pylori*** & **CagA^a^**			
		**N^b^**	**Neg/Neg**	**Pos/Neg**	**Pos/Pos**	***P*^c^**
Latent Toxoplasmosis^d^	Negative	1420	69.1	8.8	22.1	<.001
	Positive	365	52.6	15.1	32.4	
Gender	Female	873	68.4	9.0	22.6	.299
	Male	912	63.8	10.9	25.3	
Age	20—30	553	72.8	5.8	21.4	<.001
	31—40	512	68.0	10.1	21.9	
	41—50	425	63.8	10.2	25.9	
	51—60	295	51.0	18.0	31.0	
Race-ethnicity	Non-Hispanic white	750	74.8	8.3	16.8	<.001
	Non-Hispanic black	480	36.1	11.0	52.9	
	Hispanic	498	33.1	21.9	45.0	
	Other	57	30.6	19.2	50.2	
Education	No high school diploma	577	46.0	15.9	38.1	<.001
	High school diploma	590	64.1	11.4	24.5	
	More than high school	618	76.7	6.2	17.1	
Poverty-to-income ratio^d^	Not in poverty	1423	68.0	8.7	23.3	<.001
	In poverty	362	50.1	20.7	29.2	
Self-rated health	Poor	34	49.6	24.0	26.4	<.001
	Fair	282	46.7	20.2	33.1	
	Good	647	63.7	11.4	24.8	
	Very good	466	71.6	6.8	21.6	
	Excellent	356	71.5	6.8	21.7	
Hemoglobin	6.7 – 12.6	364	60.8	11.7	27.5	.583
	12.7 – 14.0	575	67.7	8.7	23.6	
	14.1 – 18.3	846	66.9	10.1	23.0	

Abbreviations: Neg, Negative; Pos, Positive.

^a^Prevalence percentages are weighted to be representative of the US population. Row percentages add to 100 except for rounding error.

^b^Numbers are unweighted numbers of participants.

^c^ *P* based on Pearson’s *χ* ^2^test.

^d^Defined as total family income divided by poverty threshold, as determined by the US Census Bureau for the year of the interview

The analysis of the relationship between *H. pylori* with CagA and cognitive functioning for the three cognitive functioning measures (SDL, SRT, and SDS) is presented in Table 2. Because performance on these cognitive measures reflects time to complete or number of trials to completion, higher scores represent worse cognitive function. Consequently, positive coefficients indicate worse cognitive functioning. Multivariate tests were only estimated for the prediction of cognitive functioning from *H. pylori* with CagA status. Multivariate tests were not performed for the relationship of cognitive functioning with the other variables in the model because they did not inform the hypothesis being tested at this stage of the analysis.

**Table 2:** Analysis of Three Measures of Cognitive Functioning in US 21 to 59 Year-Olds, NHANES III (1988–1994): Unstandardized Coefficients from Weighted OLS Regression.

		**Dependent Variable 3 SDS**		**Dependent Variable 2 SRT**		**Dependent Variable 1 SDL**	
		**b**	**95% CI**	**b**	**95% CI**	**b**	**95% CI**
	Neg/Neg	.000	[.000,.000]	.000	[.000,.000]	.000	[.000,.000]
H. pylori & CagA	Pos/Neg	.028	[-.084,.140]	3.797	[-2.814,10.408]	-.033	[-.679,.613]
	Pos/Pos	.079	[-.123,.281]	.466	[-8.755,9.687]	.700	[-.229,1.629]
Latent Toxoplasmosis^a^	Negative	.000	[.000,.000]	.000	[.000,.000]	.000	[.000,.000]
	Positive	.085	[-.018,.188]	5.654	[-5.021,16.330]	-.101	[-.699,.496]
Age		.033***	[.030,.037]	.239	[-.059,.537]	.096***	[.074,.118]
Gender	Male	.000	[.000,.000]	.000	[.000,.000]	.000	[.000,.000]
	Female	-.147**	[-.232,-.062]	13.758**	[5.460,22.056]	-.169	[-.742,.404]
	Non-Hispanic white	.000	[.000,.000]	.000	[.000,.000]	.000	[.000,.000]
Race	Non-Hispanic black	.434***	[.332,.536]	12.338**	[3.697,20.979]	1.510***	[.776,2.245]
	Hispanic	.295**	[.112,.478]	11.527**	[4.082,18.972]	2.392***	[1.471,3.313]
	Other	.165	[-.008,.337]	14.919	[-6.943,36.780]	3.271**	[1.291,5.250]
Highest grade achieved		-.089***	[-.110,-.068]	-1.300	[-2.829,.228]	-.471***	[-.587,-.356]
Poverty-to-income ratio^b^	0—3	-.137***	[-.201,-.073]	-3.716	[-8.961,1.529]	-.461	[-.947,.025]
	3—11	.010	[-.040,.059]	2.569	[-1.310,6.449]	-.224	[-.546,.098]
Self-rated Health		-.073**	[-.115,-.032]	-4.620*	[-8.424,-.817]	-.437**	[-.701,-.173]
Hemoglobin		.004	[-.023,.031]	-1.852	[-4.441,.736]	-.173	[-.420,.074]
Constant		3.105***	[2.612,3.598]	276.151***	[239.451,312.851]	11.843***	[7.210,16.476]
R2		.40		.12		.30	
N		1752		1785		1712	

Abbreviations: SDL, Serial digit learning test; SRT, Simple reaction time test; SDS, Symbol digit substitution test; Neg/Neg, *H. pylori* negative and CagA negative; Pos/Neg, *H. pylori* positive and CagA positive; Pos/Pos, *H. pylori* positive and CagA positive; PIR, Poverty-to-income ratio; *H. pylori*, *Helicobacter pylori*; CI, Confidence intervals.

^a^Positive defined as Serum toxoplasmosis antibody > = 7 IU/mL.

^b^Defined as total family income divided by poverty threshold, as determined by the US Census Bureau for the year of the interview.

* *P* <.05,

** *P* <.01,

*** *P* <.001.

Neither *H. pylori* positive with CagA negative status (*F*[3,21] = 0.32, n.s.) nor *H. pylori* positive with CagA positive status (*F*[3,21] = 0.92, n.s.) had a different relationship with cognitive functioning compared to *H. pylori* negative with CagA negative status. With non-significant multivariate results, significant univariate results can be ignored. But even without this adjustment, there were no significant univariate relationships between cognitive functioning and *H. pylori* with CagA status, as shown in the confidence intervals reported in Table 2.

**Figure 1:**
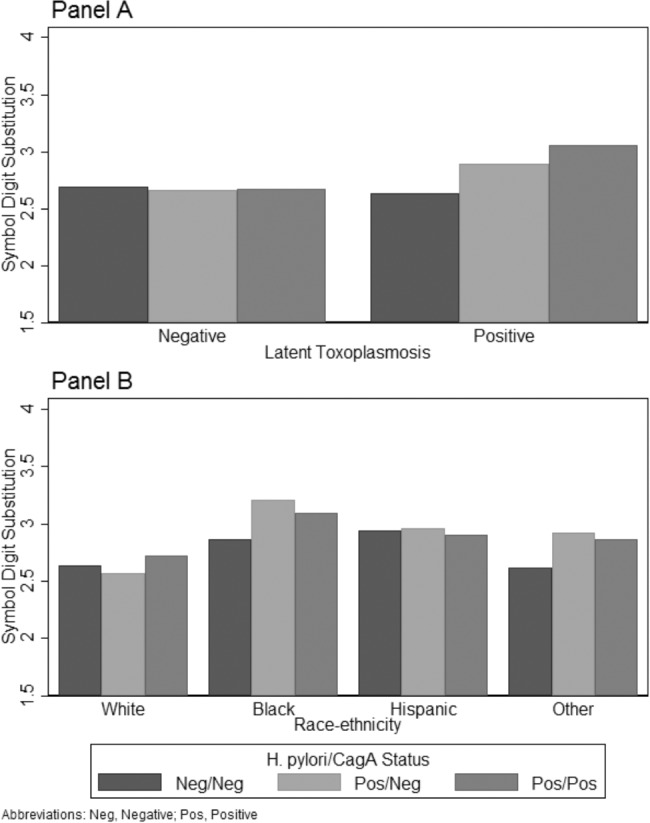
Model-based Predictions of the Symbol Digit Substitution Test Illustrating the Interaction of Latent Toxoplasmosis and Race-ethnicity with *H. pylori* and CagA. Panel A presents model-based predictions (Table 4, Model 1) of the interaction of latent toxoplasmosis with *H. pylori* and CagA on the Symbol Digit Substitution Test (SDS) controlling for age, gender, race-ethnicity, PIR, education, health, and hemoglobin. Higher SDS values indicate poorer cognitive function. Panel B presents model-based predictions (Table 4, Model 3) of the interaction of race-ethnicity with *H. pylori* and CagA on the Symbol Digit Substitution Test (SDS) controlling for latent toxoplasmosis, age, gender, PIR, education, health, and hemoglobin. Higher SDS values indicate poorer cognitive function.

**Figure 2:**
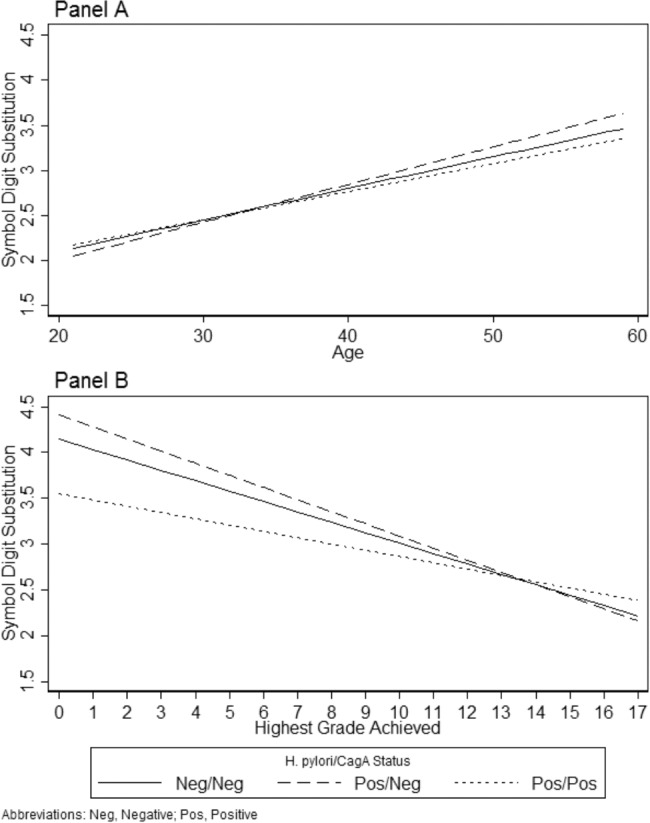
Model-based Prediction of the Symbol Digit Substitution Test Illustrating the Interaction of Age and Education with *H. pylori* and CagA. Panel A presents model-based predictions (Table 4, Model 2) of the interaction of age with *H. pylori* and CagA on the Symbol Digit Substitution Test (SDS) controlling for latent toxoplasmosis, gender, race-ethnicity, PIR, education, health, and hemoglobin. Higher SDS values indicate poorer cognitive function. Panel B presents model-based predictions (Table 4, Model 4) of the interaction of education with *H. pylori* and CagA on the Symbol Digit Substitution Test (SDS) controlling for latent toxoplasmosis, age, gender, race-ethnicity, PIR, health, and hemoglobin. Higher SDS values indicate poorer cognitive function.

**Figure 3:**
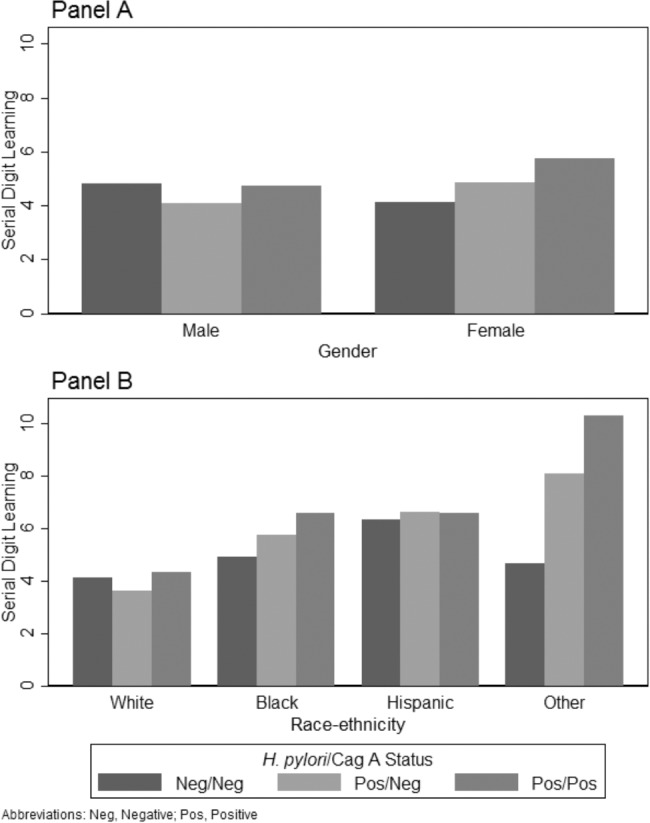
Model-based Prediction of the Serial Digit Learning Test Illustrating the Interaction of Gender and Race-Ethnicity with *H. pylori* and CagA. Panel A presents model-based predictions (Table 5, Model 1) of the interaction of gender with *H. pylori* and CagA on the Serial Digit Learning Test (SDL) controlling for latent toxoplasmosis, age, race-ethnicity, PIR, education, health, and hemoglobin. Higher values indicate poorer cognitive function on SDL. Panel B presents model-based predictions (Table 5, Model 2) of the interaction of race-ethnicity with *H. pylori* and CagA on the Serial Digit Learning Test (SDL) controlling for latent toxoplasmosis, age, gender, PIR, education, health, and hemoglobin. Higher values indicate poorer cognitive function on SDL.

**Figure 4:**
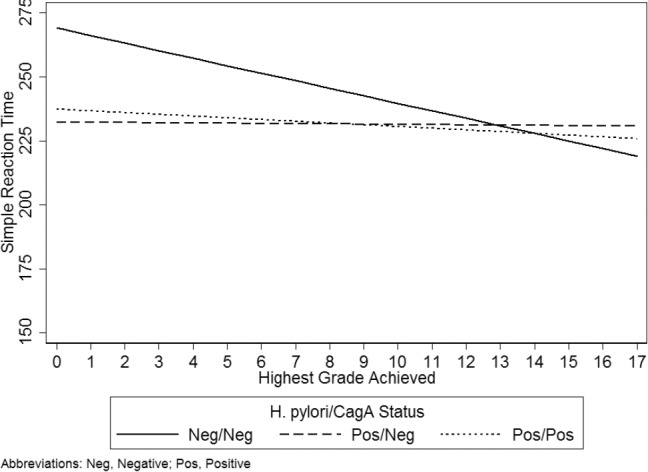
Model-based Prediction of the Simple Reaction Time Test Illustrating the Interaction of Highest Grade Achieved with *H. pylori* and CagA. Data are model-based predictions of the interaction of education with *H. pylori* and CagA on the Simple Reaction Time Test (SRT) controlling for latent toxoplasmosis, age, gender, race-ethnicity, PIR, health, and hemoglobin. Higher SRT values indicate poorer cognitive function.

Table 3 gives an overview of the second stage of analysis, which explores the interactions of *H. pylori* with latent toxoplasmosis and with the demographic covariates. The first two columns of the table give the results of the multivariate tests. Four of the multivariate tests for interactions of the demographic covariates with *H. pylori* positive with CagA negative status (gender, *F*[3,21] = 3.65, *p* = .029; black race-ethnicity, *F*[3,21] = 5.14, *p* = .008; other race-ethnicity, *F*[3,21] = 6.05, *p* = .004; education, *F*[3,21] = 3.50, *p* = .033) and another four for interactions of the demographic covariates with *H. pylori* positive with CagA positive status (latent toxoplasmosis, *F*[3,21] = 4.73, *p* = .011; age, *F*[3,21] = 7.19, *p* = .002; other race-ethnicity, *F*[3,21] = 3.20, *p* = .044; education, *F*[3,21] = 5.43, *p* = .006) were significant. The remainder of the cells indicate univariate results (interaction coefficients presented in Table 4, Table 5, and Table 6 and plots of interactions in Fig. 1, Fig. 2, Fig. 3, and Fig. 4). Significant results are indicated by “+” and “–” symbols, representing positive and negative univariate interactions between the column and row variables that accompany significant multivariate tests. For example, the Pos/Pos column under the heading “Dependent Variable 1 SDS” has + in the Latent Toxoplasmosis row, indicating a significant interaction between latent toxoplasmosis and *H. pylori* positive with CagA positive status for SDS.

**Table 3:** Direction of significant interactions of *H. pylori* and CagA with model variables, and *p* values of multivariate tests.

		***p* Values from Multivariate Tests**		**Univariate Tests**					
				**Dependent Variable 1 SDS**		**Dependent Variable 2 SRT**		**Dependent Variable 3 SDL**	
		**Pos/Neg^a^**	**Pos/Pos^b^**	**Pos/Neg^a^**	**Pos/Pos^b^**	**Pos/Neg^a^**	**Pos/Pos^b^**	**Pos/Neg^a^**	**Pos/Pos^b^**
Latent Toxoplasmosis	Positive	.202	.011		+				
Age		.219	.002		+				
Gender	Female	.029	.539					+	
Race	Non-Hispanic black	.008	.260	+				+	
	Hispanic	.613	.783						
	Other	.004	.044	+				+	
Highest grade achieved		.033	.006	-	-	-			
Poverty-to-Income Ratio		.068	.438						
Self-rated Health		.812	.376						

Notes:

^a^Pos/Neg = *H. pylori* positive and CagA negative.

^b^Pos/Pos = *H. pylori* positive and CagA positive.

Separate multivariate tests were conducted comparing respondents who were 1) H. pylori positive and CagA negative and 2) H. pylori positive and CagA positive with respondents who were negative for both H. pylori and CagA. Only significant univariate tests that were protected from alpha inflation by a significant multivariate test are reported.

Cells represent the interaction between the variable represented by the row and the column. For example, the + at the intersection of the Female row and the +/- subcolumn of the SDL column indicates an increase in SDL (worse cognitive functioning) for females who are *H. pylori* positive and CagA negative compared to females who are *H. pylori* negative and CagA negative. A minus sign indicates a negative interaction.

Significant univariate tests are only reported if there was a significant multivariate test.

**Table 4:** Multivariate Analysis of the Symbol Digit Substitution Test with Interactions of *H. pylori* in US 21 to 59 Year-Olds, NHANES III (1988–1994): Unstandardized Coefficients [95% CI] from Weighted OLS Regression.

		**Model 1 Interaction of *H. pylori* and CagA with Latent Toxoplasmosis**		**Model 2 Interaction of *H. pylori* and CagA with Age**		**Model 3 Interaction of *H. pylori* and CagA with Race-ethnicity**		**Model 4 Interaction of *H. pylori* and CagA with Highest grade achieved**	
		**b**	**95% CI**	**b**	**95% CI**	**b**	**95% CI**	**b**	**95% CI**
*H. pylori* and CagA	Neg/Neg	.000	[.000,.000]	.000	[.000,.000]	.000	[.000,.000]	.000	[.000,.000]
	Pos/Neg	-.025	[-.137,.087]	-.118	[-.325,.090]	-.070	[-.203,.064]	.596*	[.008,1.184]
	Pos/Pos	-.021	[-.159,.118]	-.347*	[-.687,-.008]	.082	[-.069,.234]	.865**	[.373,1.358]
Latent Toxoplasmosis^b^	Negative	.000	[.000,.000]	.000	[.000,.000]	.000	[.000,.000]	.000	[.000,.000]
	Positive	-.062	[-.166,.042]	.082	[-.019,.184]	.084	[-.024,.193]	.089	[-.019,.197]
Age		.033***	[.030,.037]	.031***	[.027,.035]	.033***	[.030,.036]	.033***	[.029,.036]
Race-ethnicity	Non-Hispanic white	.000	[.000,.000]	.000	[.000,.000]	.000	[.000,.000]	.000	[.000,.000]
	Non-Hispanic black	.434***	[.334,.534]	.440***	[.339,.542]	.230*	[.007,.453]	.442***	[.338,.547]
	Hispanic	.293**	[.109,.477]	.314**	[.131,.496]	.306*	[.019,.593]	.253**	[.068,.437]
	Other	.147	[-.017,.311]	.165	[-.005,.334]	-.025	[-.245,.195]	.157	[-.009,.322]
Highest grade achieved		-.089***	[-.111,-.068]	-.088***	[-.109,-.067]	-.089***	[-.110,-.068]	-.069***	[-.091,-.046]
Self-rated health		-.072**	[-.113,-.031]	-.073**	[-.115,-.031]	-.071**	[-.112,-.030]	-.072**	[-.113,-.031]
Interactions with *H. pylori* and CagA									
Latent Toxoplasmosis^b^x Pos/Neg	Negative	.000	[.000,.000]						
	Positive	.287	[-.003,.577]						
Latent Toxoplasmosis^b^x Pos/Pos	Negative	.000	[.000,.000]						
	Positive	.447*	[.050,.843]						
Age x Pos/Neg				.004	[-.002,.010]				
Age x Pos/Pos				.011*	[.002,.019]				
Race-ethnicity x Pos/Neg	Non-Hispanic white					.000	[.000,.000]		
	Non-Hispanic black					.415**	[.128,.701]		
	Hispanic					.091	[-.108,.290]		
	Other					.380*	[.033,.726]		
Race-ethnicity x Pos/Pos	Non-Hispanic white					.000	[.000,.000]		
	Non-Hispanic black					.146	[-.351,.643]		
	Hispanic					-.119	[-.512,.275]		
	Other					.174	[-.787,1.135]		
Highest grade achieved x Pos/Neg								-.045*	[-.086,-.004]
Highest grade achieved x Pos/Pos								-.064***	[-.098,-.030]
Constant		3.103***	[2.650,3.556]	3.179***	[2.680,3.679]	3.186***	[2.713,3.660]	2.835***	[2.328,3.341]
R^2^		.40		.40		.40		.40	

Abbreviations: CI, Confidence intervals; Neg/Neg, *H. pylori* negative and CagA negative; Pos/Neg, *H. pylori* positive and CagA positive; Pos/Pos, *H. pylori* positive and CagA positive.

^a^Covariates that did not have significant interactions with *H. pylori* and CagA were included in all models but not shown.

^b^Positive defined as Serum toxoplasmosis antibody > = 7 IU/mL.

* P <.05,

** P <.01,

*** P <.001.

**Table 5:** Analysis of the Serial Digit Learning Test with Interactions of *H. pylori* and CagA in US 21 to 59 Year-Olds, NHANES III (1988–1994): Unstandardized Coefficients [95%CI] from Weighted OLS Regression.

		**Model 1 Interaction of *H. pylori* and CagA with Gender**		**Model 2 Interaction of *H. pylori* and CagA with Race**	
		**b**	**95% CI**	**b**	**95% CI**
H. pylori and CagA	Neg/Neg	.000	[.000,.000]	.000	[.000,.000]
	Pos/Neg	-.700	[-1.518,.119]	-.499	[-1.205,.208]
	Pos/Pos	-.050	[-1.259,1.159]	.234	[-.627,1.095]
Gender	Male	.000	[.000,.000]	.000	[.000,.000]
	Female	-.683	[-1.410,.045]	-.282	[-.824,.260]
Race	Non-Hispanic white	.000	[.000,.000]	.000	[.000,.000]
	Non-Hispanic black	1.459***	[.734,2.183]	.804*	[.026,1.582]
	Hispanic	2.358***	[1.434,3.282]	2.228***	[1.240,3.215]
	Other	3.073**	[1.256,4.890]	.558	[-1.347,2.463]
Interactions with H. pylori and CagA					
Gender x Pos/Neg	Male	.000	[.000,.000]		
	Female	1.431*	[.222,2.640]		
Gender x Pos/Pos	Male	.000	[.000,.000]		
	Female	1.688	[-.681,4.057]		
Race x Pos/Neg	Non-Hispanic white			.000	[.000,.000]
	Non-Hispanic black			1.343*	[.195,2.491]
	Hispanic			.772	[-.724,2.268]
	Other			3.925***	[1.790,6.061]
Race x Pos/Pos	Non-Hispanic white			.000	[.000,.000]
	Non-Hispanic black			1.435	[-.036,2.906]
	Hispanic			-.002	[-.986,.982]
	Other			5.403	[-.083,10.889]
Constant		12.374***	[7.757,16.991]	12.152***	[7.861,16.442]
R^2^		.30		.31	

Abbreviations: CI, Confidence intervals; Neg/Neg, *H. pylori* negative and CagA negative; Pos/Neg, *H. pylori* positive and CagA positive; Pos/Pos, *H. pylori* positive and CagA positive. Note: Covariates that did not have significant interactions with *H. pylori* and CagA were included in all models but not shown. N = 1,755.

* *P* <.05,

** *P* <.01,

*** *P* <.001.

**Table 6:** Analysis of the Simple Reaction Time Test with Interactions of *H. pylori* and CagA in US 21 to 59 Year-Olds, NHANES III (1988–1994): Unstandardized Coefficients [95% CI] from Weighted OLS Regression.

		**Model 1 Interaction of *H. pylori* and CagA with Highest grade achieved**	
		**b**	**95% CI**
*H. pylori* and CagA	Neg/Neg	.000	[.000,.000]
	Pos/Neg	31.693*	[6.291,57.095]
	Pos/Pos	-5.039	[-42.572,32.494]
Highest grade achieved		-.677	[-2.273,.919]
Interactions with *H. pylori* and CagA			
Highest grade achieved x Pos/Neg		-2.262*	[-4.173,-.352]
Highest grade achieved x Pos/Pos		.584	[-2.635,3.803]
Constant		268.832***	[231.489,306.174]
R^2^		.12	

Abbreviations: CI, Confidence intervals; Neg/Neg, *H. pylori* negative and CagA negative; Pos/Neg, *H. pylori* positive and CagA negative; Pos/Pos, *H. pylori* positive and CagA positive. Note: Covariates that did not have significant interactions with *H. pylori* and CagA were included in model but not shown. N = 1,755.

* *P* <.05,

** *P* <.01,

*** *P* <.001.

The univariate OLS regression results for SDS are presented in Table 4, SRT in Table 5, and SDL in Table 6. In each of these tables, the inferential statistics for the significant interactions are given in the bottom half of the table, with the top half of each table showing the change in the statistics for the main effects of each predictor variable and demographic control variable in the context of that interaction. Model 1 of Table 4 presents coefficients for the significant interaction of *H. pylori* with CagA and latent toxoplasmosis, and Panel A of Fig. 1 shows the form of the interaction. The figure shows that the average SDS scores are about equal across the three *H. pylori/*CagA conditions in the absence of latent toxoplasmosis. However, respondents who are latent toxoplasmosis positive have higher average scores (i.e., worse cognitive functioning) when they are *H. pylori* positive and CagA positive compared to those who are *H. pylori* negative and CagA negative. Those who were *H. pylori* positive and CagA negative also had slightly higher average SDS scores compared to those negative for both *H. pylori* and CagA, although as the non-significant coefficient in Table 4, Model 1 indicates, the difference was not significant.

The remaining results in Table 4 show the interactions of between *H. pylori* and CagA status with age, race-ethnicity, and education for SDS. Model 2 indicates a significant interaction with age and *H. pylori* positive and CagA positive status, although Panel A of Fig. 2 shows that there was not a large substantive difference in how *H. pylori* and CagA status is related to SDS across the ages in the sample. Model 3 indicates a significant interaction with race-ethnicity and *H. pylori* positive and CagA negative status. Specifically, non-Hispanic blacks and other race-ethnicity had positive interactions indicating worse cognitive functioning for these groups relative to non-Hispanic whites when *H. pylori* is positive and CagA negative. This is illustrated in Fig. 1, Panel B where the SDS scores for whites are similar regardless of *H. pylori* and CagA status, but non-Hispanic blacks and other race-ethnicity have higher SDS scores if they are *H. pylori* positive and CagA negative. Finally, there are significant negative interactions in Model 4 between education and both *H. pylori* and CagA statuses. The form of the interactions is presented in Fig. 2, Panel B. The overall negative trend indicates education is protective of poorer cognitive functioning. However, when respondents were *H. pylori* positive and CagA positive, education was less protective (i.e., had a flatter slope) but when *H. pylori* positive and CagA negative, education was slightly more protective (i.e., had a steeper slope).

Only two demographic controls, gender and race-ethnicity, had significant univariate interactions with significant multivariate with *H. pylori* and CagA status. Model 1 of Table 5 presents the gender interaction, which is positive for both *H. pylori* and CagA statuses indicating a stronger effect on females than males. This is shown in Panel A of Fig. 3 where females have higher SDL scores (worse cognitive functioning) if they were *H. pylori* positive and CagA negative and even higher if they were *H. pylori* positive and CagA positive, although the latter effect was not statistically significant (Table 5, Model 1). Model 2 presents the race-ethnicity interaction. Again, the cognitive functioning of non-Hispanic blacks and other race-ethnicity is compromised disproportionately compared to non-Hispanic whites when *H. pylori* positive and CagA negative. The form of the interaction is presented in Fig. 3 Panel B. Although the non-Hispanic black and other race-ethnicity groups have even worse SDL scores when *H. pylori* is positive and CagA positive, these effects are not statistically significant (Table 5, Model 2).

Finally, only education had a significant interaction with *H. pylori* positive and CagA negative status for the SRT outcome (Table 6). The form of the interaction is presented in Fig. 4. The graphic indicates, again, that in the absence of *H. pylori*, education is protective of poor cognitive functioning. However, the graphic shows that in the presence of *H. pylori*, the salutary effect of education is no longer present, regardless of whether *H. pylori* is accompanied by CagA.

## Discussion

Based on data from a large, community-based sample weighted to be representative of the population of the United States, we found that an interaction between *H. pylori* infection and latent toxoplasmosis predicted decreased function on the SDL, even though we found no main effects on cognition for either *H. pylori* or latent toxoplasmosis alone. This finding suggests that the effects of certain infectious diseases on cognitive function may be synergistic, with more than one infectious disease resulting in greater cognitive deficits than either infection alone. Because *H. pylori* and latent toxoplasmosis are both common in the general population, many people are likely to have both infections. Indeed, we found that 20 percent of the subjects in this study who were seropositive for *H. pylori* were also seropositive for latent toxoplasmosis, suggesting that the interaction between *H. pylori* and latent toxoplasmosis on cognition could be a considerable personal and public-health problem. Our study was not designed to determine how seropositivity of one infection might affect seropositivity of the other. However, consistent with prior studies on latent toxoplasmosis [17] and *H. pylori* [9], we found that the prevalences of both infectious diseases are associated with overlapping sociodemographic conditions including PIR, age, level of education, and race-ethnicity, suggesting common environmental risk factors for both *H. pylori* and latent toxoplasmosis. It is also possible that the burden of having one infectious disease lessens the ability to resist infection from another infectious disease.

In addition to the finding of an interaction between *H. pylori* and latent toxoplasmosis on cognition, we also found an interaction between *H. pylori* and the non-Hispanic Black race-ethnicity category on cognition, broadly consistent with numerous previously reported well described health disparities according to ethnic background in the United States [33]. We found additional interactions between *H. pylori* and increasing age and an inverse association between *H. pylori* and educational attainment on cognitive function. That is, people seropositive for *H. pylori* with comparatively lower education were more likely to have cognitive deficits compared to people with comparatively higher education who were also seropositive for *H. pylori* even when controlling for educational attainment. These interactions suggest that particular groups may be more likely to manifest the negative cognitive effects associated with these infections. In that we found these effects in young to middle-aged adults aged 20 to 59 years, it is possible that long-term infection with *H. pylori* could contribute to not only age-related cognitive decline but perhaps atypical cognitive aging including dementia. In this regard, Malaguarnera et al. [10] compared healthy volunteers ranging in age from 60 to 81 years to patients with Alzheimer’s disease and patients with vascular dementia and found that *H. pylori* IgG levels were highest in the vascular-dementia group but that levels in the Alzheimer’s group were also significantly higher than the control group. While the mechanism for the association between *H. pylori* seropositivity and dementia is unknown, Kountouras et al. [34] demonstrated increased five-year survival in those patients with Alzheimer’s receiving treatment to eradicate *H. pylori*. Taken together, these findings suggest that it could be important to eradicate *H. pylori* at young ages given that we found some evidence of its detrimental effect on cognition even in young to middle-age adults. While Katan et al. [1] found that infectious disease burden was associated with greater likelihood of cognitive impairment in older adults, we found that the deficits in cognitive function associated with certain infectious diseases may start at a younger age.

The seroprevalence of *H. pylori* varied according to sociodemographic variables. We found that the seroprevalence of *H. pylori* infection was significantly higher in subjects with lower PIR levels and in those with less educational attainment. In addition, there was a much higher seroprevalence of *H. pylori* in those participants who self-rated their overall health as “poor” or “fair” compared to those who self-rated their health as “good”, “very good”, or “excellent”. Further, the oldest age group, 51 to 60 years, had an *H. pylori* seroprevalence of more than twice that of the youngest age group, whose age ranged from 20 to 30 years suggesting older age might increase risk of infection because of longer potential exposure although this could also reflect a cohort effect. Although the seroprevalence rates of *H. pylori* associated with sociodemographic variables reported in other studies using the NHANES III dataset, such as the study by Chen and Blaser [23], are slightly different from those we found, the differences likely reflect variability in the age range studied and limited sample with data on both latent toxoplasmosis and anti-CagA.

Although we found no main effects of *H. pylori* or latent toxoplasmosis on any of the measures available for cognitive function, there were main effects of race-ethnicity, educational attainment, PIR, and self-rated health. Our findings for the main effects of these covariates as they relate to cognitive function were consistent with prior studies [9, 17, 20].

The underlying mechanism for cognitive impairment related to *H. pylori* infection is unclear. Because *H. pylori* is associated with gastritis, it may increase the risk for vitamin B-12 deficiency and the subsequent increase of homocysteine [35], although Kountouras et al., [36] found higher homocysteine concentrations in anemic controls compared to age-matched patients with mild cognitive impairment (MCI). They also found, however, a much higher prevalence of *H. pylori* infection in the MCI group (88.9 percent vs. 48.6 percent) compared to the anemic control group. Anti*-H. pylori* IgG serum concentrations also correlated with a measure of cognition in the MCI group with *H. pylori* infection. The effects of iron deficiency may also be important for cognition, at least during development. Muhsen et al. [37] found that *H. pylori* infection was associated with a decrease of 4 to 6 IQ points in children with *H. pylori* compared to children without *H. pylori* infection, a difference that may be related to anemia and iron deficiency in that *H. pylori* appears to be associated with iron deficiency. For example, Cardenas et al. [26] found that *H. pylori* infection resulted in a 40 percent increase in iron deficiency. Therefore, it is possible that *H. pylori* may have a secondary effect on the brain by increasing homocysteine that in turn could compromise cerebrovascular health or perhaps by depleting needed nutrients [5]. Given the potential for the presence of iron deficiency to confound the association between cognition and *H. pylori*, we included hemoglobin as a covariate in the current study. Finally, it is also possible that the presence of *H. pylori* infection and the concomitant immunological response could explain the cognitive deficits associated with *H. pylori* infection, consistent with the findings of Mawanda and Wallace [3] showing associations between many different infections and Alzheimer’s disease. As for a possible connection between *H. pylori* infection and AD pathology, Wang et al. [38] demonstrated that *H. pylori* was not only associated with cognitive impairment in rats but was also associated with the promotion of Aβ_42_ production and with higher levels of presenilin-2, although such an association has not been shown in humans and it is still unclear whether *H. pylori* is directly associated with neuronal integrity [39].

Several possible mechanisms exist by which latent toxoplasmosis could affect cognitive function. Latent toxoplasmosis could alter dopamine metabolism [40], affect gene expression [41], or directly alter neuronal function [42].

Like Beydoun et al. [9], we also found that an interaction between *H. pylori* and race-ethnicity predicted cognitive function and that an interaction between sex and *H. pylori* predicted performance on the SDL task, with females appearing to be more susceptible on this task to the effects of *H. pylori* seropositivity than were males. In addition, we found interactions between *H. pylori* and age and between *H. pylori* and educational attainment predicted cognitive function.

While this study has several strengths including the use of a large dataset weighted to be representative of the US population, objective measure of exposure, and objective outcome measures, several limitations to this study require consideration when interpreting these findings. The cross-sectional design precludes determination of cause and effect. Instead of the interaction between *H. pylori* seropositivity and latent toxoplasmosis causing deficits in cognition, it is possible that cognitive deficits themselves may result in an increased liability to *H. pylori* infection and concomitant latent toxoplasmosis. Because the study design did not include randomization, residual confounding could be present, despite our attempts to statistically control for confounding. One of the control variables we used was self-rated health, which may not accurately assess health status [43]. Furthermore, the cognitive outcomes in this study assessed only simple reaction time, short-term memory, and attention, leaving many areas of cognitive function unevaluated. If anything, though, this would make our findings of the associations between the interactions with *H. pylori* and latent toxoplasmosis, age, race-ethnicity, and educational attainment conservative but incomplete. The interactions with *H. pylori* could affect other aspects of cognition such as executive function, other types of memory, and processing speed, factors that we were unable to evaluate.

In conclusion and within the context of the limitations of this study, we found that the interaction between *H. pylori* seroprevalence and latent toxoplasmosis predicted cognitive dysfunction in young and middle-age adults, despite the lack of main effects on cognition from either *H. pylori* infection or latent toxoplasmosis alone. This interaction possibly represents a synergistic effect between these two common infectious diseases on cognition. We also found that interactions between *H. pylori* seroprevalence and age, race-ethnicity, and educational attainment predicted cognitive function. As such, *H. pylori* appears to adversely affect cognitive ability in certain groups.
